# Response to glecaprevir/pibrentasvir in HIV/HCV-coinfected patients in clinical practice

**DOI:** 10.1093/jac/dkad278

**Published:** 2023-09-06

**Authors:** Alejandro Gonzalez-Serna, Anaïs Corma-Gomez, Francisco Tellez, Diana Corona-Mata, María Jose Rios-Villegas, Dolores Merino, Carlos Galera, Antonio Ramon Collado-Romacho, Ignacio De Los Santos, Josep Cucurull, Marta Santos, Sofía García-Martín, Antonio Rivero, Luis Miguel Real, Juan Macias

**Affiliations:** Infectious Diseases and Microbiology Unit, Hospital Universitario de Valme, Avda Bellavista s/n, 41014 Seville, Spain; Departamento de Fisiología, Facultad de Farmacia, Universidad de Sevilla, Sevilla, Spain; Instituto de Biomedicina de Sevilla (IBiS), Sevilla, Spain; Centro de Investigación Biomédica en Red de Enfermedades Infecciosas (CIBERINFEC), Madrid, Spain; Grupo Estudio Hepatitis Víricas (GEHEP) de la SEIMC, GEHEP-001, Sevilla, Spain; Infectious Diseases and Microbiology Unit, Hospital Universitario de Valme, Avda Bellavista s/n, 41014 Seville, Spain; Instituto de Biomedicina de Sevilla (IBiS), Sevilla, Spain; Centro de Investigación Biomédica en Red de Enfermedades Infecciosas (CIBERINFEC), Madrid, Spain; Grupo Estudio Hepatitis Víricas (GEHEP) de la SEIMC, GEHEP-001, Sevilla, Spain; Grupo Estudio Hepatitis Víricas (GEHEP) de la SEIMC, GEHEP-001, Sevilla, Spain; UGC Enfermedades Infecciosas, Departamento de Medicina, Universidad de Cádiz, Hospital Universitario de Puerto Real, Cádiz, Spain; Grupo Estudio Hepatitis Víricas (GEHEP) de la SEIMC, GEHEP-001, Sevilla, Spain; Infectious Diseases Unit. Maimonides Institute of Biomedical Research of Córdoba (Instituto Maimónides de Investigación Biomédica de Córdoba IMIBIC), Reina Sofía University Hospital of Córdoba, University of Córdoba, Córdoba, Spain; Grupo Estudio Hepatitis Víricas (GEHEP) de la SEIMC, GEHEP-001, Sevilla, Spain; Hospital Universitario Virgen Macarena, Unidad de Enfermedades Infecciosas, Sevilla, Spain; Grupo Estudio Hepatitis Víricas (GEHEP) de la SEIMC, GEHEP-001, Sevilla, Spain; Hospital Juan Ramón Jiménez, Unidad de Enfermedades Infecciosas, Huelva, Spain; Grupo Estudio Hepatitis Víricas (GEHEP) de la SEIMC, GEHEP-001, Sevilla, Spain; Hospital Universitario Virgen de la Arrixaca, Unidad de Medicina Interna, Murcia, Spain; Grupo Estudio Hepatitis Víricas (GEHEP) de la SEIMC, GEHEP-001, Sevilla, Spain; Unidad de Enfermedades Infecciosas, Hospital Universitario Torrecárdenas, Almería, Spain; Grupo Estudio Hepatitis Víricas (GEHEP) de la SEIMC, GEHEP-001, Sevilla, Spain; CIBERINFEC, Hospital Universitario de La Princesa, Madrid, Spain; Grupo Estudio Hepatitis Víricas (GEHEP) de la SEIMC, GEHEP-001, Sevilla, Spain; Medicina Interna, Fundació Salut Empordà, Figueres, Spain; Infectious Diseases and Microbiology Unit, Hospital Universitario de Valme, Avda Bellavista s/n, 41014 Seville, Spain; Instituto de Biomedicina de Sevilla (IBiS), Sevilla, Spain; Centro de Investigación Biomédica en Red de Enfermedades Infecciosas (CIBERINFEC), Madrid, Spain; Grupo Estudio Hepatitis Víricas (GEHEP) de la SEIMC, GEHEP-001, Sevilla, Spain; Grupo Estudio Hepatitis Víricas (GEHEP) de la SEIMC, GEHEP-001, Sevilla, Spain; UGC Microbiología, Hospital Universitario de Puerto Real, Cádiz, Spain; Grupo Estudio Hepatitis Víricas (GEHEP) de la SEIMC, GEHEP-001, Sevilla, Spain; Infectious Diseases Unit. Maimonides Institute of Biomedical Research of Córdoba (Instituto Maimónides de Investigación Biomédica de Córdoba IMIBIC), Reina Sofía University Hospital of Córdoba, University of Córdoba, Córdoba, Spain; Infectious Diseases and Microbiology Unit, Hospital Universitario de Valme, Avda Bellavista s/n, 41014 Seville, Spain; Instituto de Biomedicina de Sevilla (IBiS), Sevilla, Spain; Centro de Investigación Biomédica en Red de Enfermedades Infecciosas (CIBERINFEC), Madrid, Spain; Grupo Estudio Hepatitis Víricas (GEHEP) de la SEIMC, GEHEP-001, Sevilla, Spain; Departamento de Especialidades Quirúrgicas, Bioquímica e Inmunología, Facultad de Medicina, Universidad de Málaga, Málaga, Spain; Infectious Diseases and Microbiology Unit, Hospital Universitario de Valme, Avda Bellavista s/n, 41014 Seville, Spain; Instituto de Biomedicina de Sevilla (IBiS), Sevilla, Spain; Centro de Investigación Biomédica en Red de Enfermedades Infecciosas (CIBERINFEC), Madrid, Spain; Grupo Estudio Hepatitis Víricas (GEHEP) de la SEIMC, GEHEP-001, Sevilla, Spain; Departamento de Medicina, Universidad de Sevilla, Sevilla, Spain

## Abstract

**Objectives:**

HIV infection has been associated with lower rates of sustained viral response (SVR) with direct-acting antivirals (DAAs). There are few data on glecaprevir/pibrentasvir (G/P) in HIV/HCV coinfection outside clinical trials.

**Methods:**

The HEPAVIR-DAA cohort, which recruits HIV/HCV-coinfected patients (NCT02057003) and the GEHEP-MONO cohort (NCT02333292), including HCV-monoinfected individuals, are two concurrent ongoing multicentre cohorts of patients receiving anti-HCV treatment. Patients starting G/P included in those cohorts were analysed. Overall SVR (ITT), discontinuations due to adverse effects, and dropouts were evaluated and compared between both cohorts.

**Results:**

Of the 644 patients who started G/P with evaluable SVR, 132 were HIV/HCV coinfected. Overall SVR rates were 487/512 (95.1%) in HCV-monoinfected patients versus 126/132 (95.5%) in HIV/HCV-coinfected patients (*P* = 1.000). One patient (0.8%) relapsed, and another (0.8%) discontinued treatment due to side effects. SVR to 8 or 12 weeks of treatment with G/P was similar in HIV/HCV-coinfected versus HCV-monoinfected patients. The main reason for not reaching SVR among HIV/HCV-coinfected patients was premature dropout linked to active drug use.

**Conclusions:**

G/P in HIV/HCV coinfection was highly effective and tolerable in clinical practice. SVR to 8 or 12 weeks of treatment with G/P was similar in HIV/HCV-coinfected compared with HCV-monoinfected patients but active drug use is still a barrier to reach HCV microelimination.

## Introduction

Glecaprevir/pibrentasvir (G/P) has demonstrated high efficacy and tolerability in a variety of settings.^1–4^ Clinical trials have shown that G/P is well tolerated and highly effective, including patients with compensated cirrhosis, with an overall cure rate of 98%.^5^ Current guidelines recommend 8 weeks of G/P treatment for some patients without the need for baseline resistance testing.^6^ However, information on G/P treatment in people living with HIV (PLWH) is still scarce.

In PLWH, G/P for 8 weeks in non-cirrhotic patients and 12 weeks in cirrhotic patients was highly effective in one clinical trial.^7^ Unfortunately, G/P clinical trial data for 8 weeks of treatment in cirrhotic PLWH are not yet available. A *post hoc* analysis of G/P in patients in clinical trials and real-world studies found that it was well tolerated in different populations of patients.^8^ However other DAAs, such as sofosbuvir/ledipasvir, have shown higher relapse rates for 8 week treatment compared with 12 week treatment among PLWH.^9^ Also, the higher proportion of active drug users among coinfected HIV/HCV patients compared with HCV-monoinfected patients could result in reduced sustained viral response (SVR) rates.^4,10,11^ Finally, there are few data on the efficacy of G/P together with combinations of ART under real-world conditions of use.

Due to all of these, SVR rates for G/P in PLWH could be lower than in HCV-monoinfected patients. Therefore, our aim was to evaluate the efficacy and tolerability of G/P for PLWH compared with HCV-monoinfected patients in daily clinical practice.

### Patients and methods

#### Patients and study design

The HEPAVIR-DAA cohort (NCT02057003), which includes HIV/HCV-coinfected patients, and the GEHEP-MONO cohort (NCT02333292), which recruits HCV-monoinfected individuals, are two concurrent ongoing prospective multicentre cohorts of patients receiving DAA combinations prescribed in clinical practice, outside clinical trials. The patients included in these cohorts with chronic HCV infection who started G/P were included in the present analysis. Patients taking at least one dose of the combination were eligible. Individuals were excluded if they underwent a liver transplant before reaching the date of SVR evaluation. Cirrhosis was diagnosed with a liver biopsy, if liver stiffness ≥12.5 kPa was found, or when the patients had developed a previous hepatic decompensation.

### Medications and follow-up

G/P was used as prescription medication to treat HCV infection in routine clinical practice in the cohorts. The standard duration of the combination G/P was 8 weeks for treatment-naive patients without cirrhosis.^12^ Since 2019, treatment-naive patients with compensated cirrhosis could be treated for 8 weeks. Achievement of plasma HCV RNA below the detection limit 12 weeks after the end of G/P therapy was defined as SVR.

Active drug use was defined as ongoing drug use for 12 months before starting G/P. Drug use was self-reported and assessed by physician-driven interview during clinical visits. Individuals using cannabis alone were not classified as active drug users. In Spain, opioid agonist therapy (OAT) is managed by drug addiction facilities. Data on the use of OAT among patients included in the cohorts were recorded.

### Statistical analyses

The efficacy of therapy was assessed by the SVR rate. Discontinuations due to adverse effects, dropouts and virological failures (breakthrough or relapse) were analysed in patients according to drug use. SVR rates were estimated by an ITT analysis, considering all missing data at the date of the SVR assessment as failures. Discontinuations due to adverse effects, virological failure and dropouts were also evaluated. SVR rates were estimated for the 8 week treatments and for the 12 week treatments. In addition, a per-protocol (PP) analysis approach was used to calculate the SVR rates, excluding patients discontinuing therapy for non-treatment-related reasons.

Continuous variables were expressed as median (IQR) and categorical variables as number (%). The chi-squared test was used to compare proportions between treatment groups. The Mann–Whitney *U*-test or Kruskal–Wallis test were applied for comparisons of continuous variables among groups. Data were analysed using IBM SPSS 28.0 version (IBM Corporation, Somers, NY, USA) and STATA 16.0 (StataCorp LP, College Station, TX, USA).

### Ethics

Both the study design and development complied with the Declaration of Helsinki and was approved by the local Ethics Committee of the Hospital Universitario Virgen de Valme (Seville). All patients gave their written informed consent to participate in the study.

## Results

### Baseline characteristics of the patients

**Figure 1.**
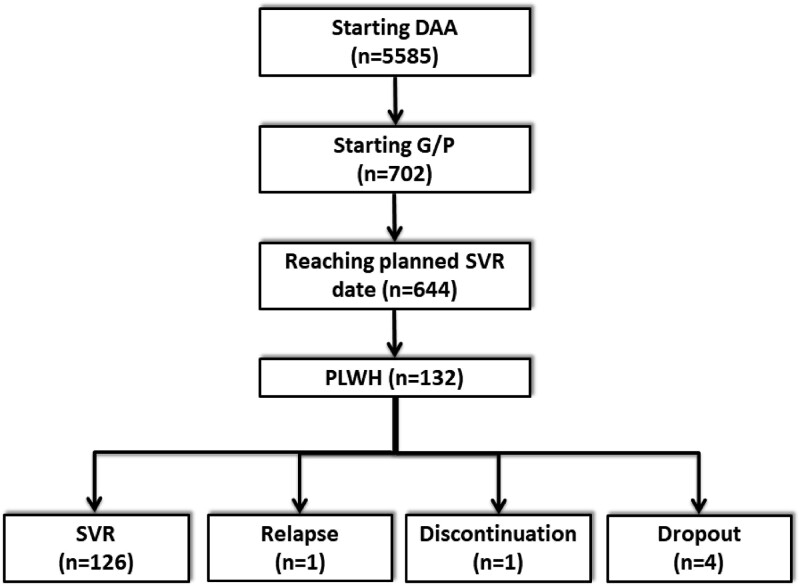
Flow chart of patients.

Overall, 5585 patients included in the cohorts had started interferon-free DAA combinations since November 2017. Of these, 644 patients started G/P and reached the date of SVR evaluation (Figure 1). Of these, 132 (20.5%) were HIV/HCV coinfected. All these patients had undetectable HIV-RNA viral load at treatment onset. Their characteristics at the date of starting G/P are summarized in Table 1.

**Table 1.** Baseline characteristics

Characteristics (*N* = 644)	HCV-monoinfected (*N* = 512)	HIV/HCV-coinfected (*N* = 132)	*P* value
Male sex, *n* (%)	292 (57)	88 (66.7)	0.047
Age, years, median (IQR)	51.2 (46.8–55.1)	50.2 (45.7–52.4)	0.434
CD4, cells/mL, median (IQR)	—	560 (350–853)	—
PWID, *n* (%)	281 (54.9)	94 (71.2)	0.001
OAT, *n* (%)	87 (17)	29 (22)	0.204
Active drug use, *n* (%)	59 (11.5)	23 (17.4)	0.079
HCV genotype 3, *n* (%)	92 (18.7)	27 (20.9)	0.615
Cirrhosis^a^, *n* (%)	38 (7.4)	15 (11.4)	0.155
G/P scheduled for 8 weeks, *n* (%)	470 (91.8)	106 (80.3)	<0.001
Retreatment^b^, *n* (%)	53 (10.4)	24 (18.2)	0.023
Liver stiffness, kPa, median (IQR)	6.5 (5.3–8.6)	6.8 (5.3–9.3)	0.355

Cirrhosis was diagnosed with a liver biopsy showing fibrosis stage 4, or with liver stiffness ≥12.5 kPa, or with a previous decompensation of cirrhosis.

Previous treatment with PEG-interferon plus ribavirin; PWID, people with current or past injecting drug use. Among HIV/HCV-coinfected patients with cirrhosis, 3/15 (20%) received 8 weeks of treatment.

### Response to treatment

Virological and non-virological outcomes according to HIV coinfection are summarized in Table 2. We found no statistically significant differences in response to treatment according to HIV coinfection. Among HIV/HCV-coinfected patients, 126 [95.5% (95% CI: 92%–99%)] patients achieved SVR (ITT analysis). No individuals showed virological breakthrough before the end of treatment. One (0.8%) patient showed relapse after the end-of-treatment response and one (0.8%) patient showed discontinuation due to adverse events (cirrhotic patient with Child–Pugh score B7 who developed hepatic encephalopathy). Five (3.8%) patients prematurely discontinued treatment. One patient who prematurely discontinued treatment achieved SVR.

**Table 2.** Virological and non-virological outcomes according to HIV coinfection

Outcome (*N* = 132)	HCV-monoinfected (*N* = 512)	HIV/HCV-coinfected (*N* = 132)	*P* value
Discontinuation due to adverse events, *n* (%)	0 (0)	1 (0.8)	0.049
Dropout, *n* (%)	30 (5.9)	5^a^ (3.8)	0.517
Viral breakthrough, *n* (%)	0 (0)	0 (0)	—
Viral relapse, *n* (%)	1 (0.2)	1 (0.8)	0.301
SVR ITT, *n* (%)	487 (95.1)	126 (95.5)	1.000

One patient with active drug use achieved SVR despite prematurely discontinuing treatment.

### SVR response according to drug use

The SVR rates were 87% (20/23) for PLWH with active drug use, compared with 97.2% (106/109) for PLWH without active drug use (*P* = 0.065). Among PLWH with active drug use, 4/23 (17.44%) patients prematurely discontinued treatment, but one of them achieved SVR. After excluding those patients who discontinued therapy for reasons not related to treatment (PP analysis), SVR rates were 100% (19/19) for PLWH with active drug use.

### SVR response according to ART

We found no differences among SVR rates according to ART (Table 3). Reasons for not achieving SVR for patients on abacavir/lamivudine/dolutegravir (ABC/3TC/DTG) were: one patient relapsed; and three patients prematurely discontinued treatment. One of these achieved SVR despite discontinuing treatment. In addition, one patient on tenofovir alafenamide/emtricitabine/elvitegravir-cobicistat (TAF/FTC/EVG-c) and another patient on tenofovir disoproxil fumarate/emtricitabine did not achieve SVR due to premature discontinuation in both cases. Finally, one patient on dolutegravir/lamivudine discontinued treatment due to adverse events.

**Table 3.** SVR (ITT) according to ART (*N* = 132)

ART, *n* (%)	N	SVR
ABC/3TC/DTG	48	45/48 (93.8)
TDF/FTC/RPV	22	21/22 (95.5)
TAF/FTC/EVG-c	13	12/13 (92.3)
ABC/3TC + RPV	9	9/9 (100)
TDF/FTC + DTG	8	8/8 (100)
TDF/FTC/EVG-c	7	7/7 (100)
ABC/3TC + RAL	5	5/5 (100)
TDF/FTC + RAL	4	4/4 (100)
DTG/3TC	4	3/4 (75)
TAF/FTC/BIC	1	1/1 (100)
TAF/FTC/DRV-c	1	1/1 (100)
TAF/FTC + DTG	1	1/1 (100)
TAF/FTC/RPV	1	1/1 (100)
ABC/3TC + NVP	1	1/1 (100)
ABC/3TC + DRV-c	1	1/1 (100)
TDF/FTC/EFV	1	1/1 (100)
DTG/RPV	1	1/1 (100)
DRV-c + MVC	1	1/1 (100)
DRV-c	1	1/1 (100)
No ART	2	2/2 (%)

There was no statistical difference among ART (*P* = 0.156). One patient on other combinations showed discontinuation due to adverse events (cirrhotic patient with Child–Pugh score B7 who developed hepatic encephalopathy). ABC, abacavir; 3TC, lamivudine; DTG, dolutegravir; FTC, emtricitabine; TAF, tenofovir alafenamide; TDF, tenofovir disoproxil fumarate; EVG-c, elvitegravir-cobicistat; FTC, emtricitabine; RPV, rilpivirine; RAL, raltegravir; BIC, bictegravir; DRV-c, darunavir-cobicistat; EFV, efavirenz; MVC, maraviroc; NVP: nevirapine.

### SVR response according to cirrhosis and genotype

Among PLWH without cirrhosis, 112/117 (95.7%) achieved SVR (ITT), compared with 14/15 (93.3%) among those with cirrhosis (*P* = 0.522) (Table 4). A 12 week treatment was planned for 12/15 (80%) patients with cirrhosis and for 14/117 (12%) without cirrhosis. SVR (ITT) was achieved by 24/27 (88.9%) patients with genotype 3, compared with 99/102 (97.1%) without genotype 3 (*P* = 0.106). Among PLWH with cirrhosis without genotype 3, 12/12 (100%) achieved SVR. Two of three PLWH with cirrhosis and genotype 3 reached SVR after 12 weeks of treatment and one showed discontinuation due to adverse events (cirrhotic patient with Child–Pugh score B7 who developed hepatic encephalopathy) with a planned duration of treatment of 12 weeks.

**Table 4.** SVR (ITT) to G/*P* in HIV/HCV-coinfected patients

Variable (*N* = 132)	*n*	SVR, %	*P* value
Sex			
Male	88	93.2	0.380
Female	44	96.6	
Age, years			
>51	57	94.7	0.452
≤ 51	75	96	
Receiving OAT			
Yes	29	100	0.338
No	103	94.2	
Active drug use^a^			
Yes	23	87	0.065
No	109	97.2	
HCV genotype 3^b^			
Yes	27	88.9	0.106
No	102	97.1	
Cirrhosis^c^			
Yes	15	93.3	0.522
No	117	95.7	
G/P treatment duration, weeks			
8	106	96.2	0.337
12	26	92.3	
Baseline HCV RNA^d^, IU/mL			
<1.5 × 10^6^	63	96.8	0.680
≥ 1.5 × 10^6^	63	93.7	

Self-reported recent drug use (<12 months) parenterally and/or orally/inhaled.

Genotype was not available in 3 HIV/HCV-coinfected patients.

Cirrhosis was diagnosed with a liver biopsy showing fibrosis stage 4, or with liver stiffness ≥12.5 kPa, or with a previous decompensation of cirrhosis.

Baseline HCV RNA was not available in 6 HIV/HCV-coinfected patients.

## Discussion

In daily clinical practice, HIV/HCV-coinfected patients achieve high overall SVR rates, greater than 95%, to G/P. The response to G/P was similar in HIV/HCV-coinfected patients compared with HCV-monoinfected patients. The main reason for not reaching SVR among HIV/HCV-coinfected patients was voluntary dropout linked to active drug use.

Overall, the high SVR rate found here for HIV/HCV coinfection in clinical practice, irrespective of treatment duration or cirrhosis status, is in agreement with a pooled analysis of ongoing, multinational, post-marketing observational studies,^13^ and also consistent with the G/P registration trials, the EXPEDITION-8 trial and the EXPEDITION-2 trial.^14,15^ In terms of duration of treatment, no large differences were observed for SVR between patients treated for 8 or 12 weeks, which is consistent with previous meta-analysis results and the EXPEDITION-8 trial results.^14,16^ Therefore, and in agreement with other studies, our results support a shorter duration of G/P treatment, which could lead to greater savings in time and resources, both in terms of healthcare visits and downstream costs.^17,18^

ART regimens used here did not have an impact on G/P response. G/P has a favourable drug–drug interaction profile with many ARTs. Thus, G/P can be combined safely with integrase inhibitors, NRTIs, and the NNRTI rilpivirine.^15^ Exposures of G/P may increase by coadministration with ritonavir- or cobicistat-containing regimens and should be avoided with ART regimens that induce P-glycoprotein and cytochrome P450, such as efavirenz.^19,20,21^ In the present study, 24 PLWH were prescribed by their caring physician a regimen that included cobicistat as booster. Twenty of them received cobicistat-boosted elvitegravir. Four PLWH were prescribed cobicistat-boosted darunavir. The use of boosted PIs along with G/P is contraindicated. Fortunately, none of them reported suffering adverse events leading to G/P discontinuation.

Active drug use among patients with HIV/HCV coinfection was the only factor that showed a trend to an association with reduced SVR rates. This agrees with recent studies on HCV-infected individuals in real-world clinical practice.^4,22^ Henceforth, DAA treatment for active drug users must be complemented with some sort of strategy to ensure adherence. Numerous potential interventions to improve the retention of healthcare for drug users have been described, such as the co-location of OAT and DAA therapy,^23^ peer support or patient navigator,^24^ and cash incentives.^25^

This study has certain limitations. First, as an observational clinical practice study, some ARTs contraindicated with GP were used. We did not document any serious adverse events or those involving discontinuation in these patients. However, we cannot rule out that other milder adverse events went unnoticed. Second, 8 or 12 week treatment durations were decided by the treating physician at each moment, so the easiest patients to treat have been selected to receive 8 week treatment as we show in our results. Third, drug use was self-reported and thus, it was likely underestimated. Fourth, our study includes a small number of HIV/HCV-coinfected patients with cirrhosis treated for 8 weeks. All of them achieved SVR. For compensated cirrhosis in PLWH and HCV, current recommendations on G/P for 8 weeks in HCV-monoinfected patients with cirrhosis should be assumed. These study data are insufficient to support that recommendation. On the other hand, this is a study with a high number of HIV/HCV-coinfected patients whose responses are compared with HCV-monoinfected patients managed by the same clinical units. In addition, with a sample size of 132 and an SVR rate of 95.5% (95% CI: 92%–99%), a statistical precision (ω) of 3.3% is achieved, which is another strength of the study.

In conclusion, G/P in HIV/HCV coinfection was highly effective and tolerable in clinical practice. SVR to 8 or 12 weeks of treatment with G/P was similar in HIV/HCV-coinfected compared with HCV-monoinfected patients. Of note, the 8 weeks of G/P treatment may be important in areas and groups where microelimination will be more difficult to achieve or where the coinfected population is still large. SVR rates and tolerability were not influenced by the ART combinations used in this clinical practice subset. However, active drug use in HIV/HCV coinfection is a barrier to reach the HCV microelimination goal in this population that needs to be tackled.
